# Developmental exposure to silver nanoparticles leads to long term gut dysbiosis and neurobehavioral alterations

**DOI:** 10.1038/s41598-021-85919-7

**Published:** 2021-03-22

**Authors:** Zhen Lyu, Shreya Ghoshdastidar, Karamkolly R. Rekha, Dhananjay Suresh, Jiude Mao, Nathan Bivens, Raghuraman Kannan, Trupti Joshi, Cheryl S. Rosenfeld, Anandhi Upendran

**Affiliations:** 1 Department of Electrical Engineering and Computer Science, University of Missouri, Columbia, MO 65212 USA; 2 Department of Biomedical, Biological and Chemical Engineering, University of Missouri, Columbia, MO 65211 USA; 3 Department of Radiology, University of Missouri, Columbia, MO 65212 USA; 4 Department of Biomedical Sciences, University of Missouri, Columbia, MO 65212 USA; 5 DNA Core Facility, University of Missouri, Columbia, MO 65212 USA; 6 Department of Health Management and Informatics, University of Missouri, Columbia, MO 65212 USA; 7 MU Institute of Data Science and Informatics, University of Missouri, Columbia, MO 65212 USA; 8 Bond Life Sciences Center, University of Missouri, Columbia, MO 65212 USA; 9 Genetics Area Program, University of Missouri, Columbia, MO 65212 USA; 10 Thompson Center for Autism and Neurobehavioral Disorders, University of Missouri, Columbia, MO 65212 USA; 11 Department of Medical Pharmacology & Physiology, University of Missouri, Columbia, MO 65212 USA; 12 MU-Institute of Clinical and Translational Science, University of Missouri, Columbia, MO 65212 USA

**Keywords:** Developmental biology, Nanoscience and technology

## Abstract

Due to their antimicrobial properties, silver nanoparticles (AgNPs) are used in a wide range of consumer products that includes topical wound dressings, coatings for biomedical devices, and food-packaging to extend the shelf-life. Despite their beneficial antimicrobial effects, developmental exposure to such AgNPs may lead to gut dysbiosis and long-term health consequences in exposed offspring. AgNPs can cross the placenta and blood–brain-barrier to translocate in the brain of offspring. The underlying hypothesis tested in the current study was that developmental exposure of male and female mice to AgNPs disrupts the microbiome–gut–brain axis. To examine for such effects, C57BL6 female mice were exposed orally to AgNPs at a dose of 3 mg/kg BW or vehicle control 2 weeks prior to breeding and throughout gestation. Male and female offspring were tested in various mazes that measure different behavioral domains, and the gut microbial profiles were surveyed from 30 through 120 days of age. Our study results suggest that developmental exposure results in increased likelihood of engaging in repetitive behaviors and reductions in resident microglial cells. Echo-MRI results indicate increased body fat in offspring exposed to AgNPs exhibit. *Coprobacillus* spp., *Mucispirillum* spp., and *Bifidobacterium* spp. were reduced, while *Prevotella* spp., *Bacillus* spp., *Planococcaceae*, *Staphylococcus* spp., *Enterococcus* spp., and *Ruminococcus* spp. were increased in those developmentally exposed to NPs*.* These bacterial changes were linked to behavioral and metabolic alterations. In conclusion, developmental exposure of AgNPs results in long term gut dysbiosis, body fat increase and neurobehavioral alterations in offspring.

## Introduction

Engineered nanomaterials are used for a myriad of applications due to their unique properties. At the nanoscale level, the number of surface atoms is much higher than the bulk of the material. The large number of surface atoms in metallic nanoparticles is responsible for increased reactivity and unique photophysical properties. Nanoparticles within the size domains of 1–100 nm exhibit exceptional properties compared to source metals that make them suitable for various applications, including sensors, optoelectronic materials, and drug delivery devices^1^. Among nanomaterials that have been widely used for several applications, silver nanoparticles exhibit antimicrobial and anti-fungal activity. Silver nanoparticles (AgNPs) are used for several biomedical applications and contained within consumer products that include topical wound dressings, coatings for biomedical devices, fabrics, food-packaging to extend the shelf-life and reduce potential growth of pathogenic bacteria, as dietary health supplements, and as antimicrobial coating in several children’s products^2–16^. In spite of their beneficial antimicrobial effects, long-term exposure to these particles can cause deleterious health outcomes, including metabolic alterations, immunological changes, reproductive and importantly, gut alternations and neurotoxicity related through the microbiome-gut-brain axis^16–27^. A detailed biodistribution study following AgNP exposure have reported accumulation and toxicity effects to at the site of usage and in distal organs, especially the brain^26^. The study pointed out the need of understanding the mechanisms of specific organ toxicities due to AgNP^28^. A recent report on *hazard* characterization of AgNP suggest that they may cause neuro- and reproductive toxicity, as such particles may persist for longer periods^29^. According to a recent study the intake of silver in humans could be as high as 90 μg/day^30^. It has also been estimated that a child’s potential non-dietary ingestion exposure to AgNPs when drinking formula from a sippy cup is ~ 1.53 μg Ag/Kg^31^.

AgNP from the consumer products can leach into the environment and get exposed to human and other animals via inhalation, ingestion, or dermal contact^32^. Among these exposures, dietary ingestion is considered the primary route of exposure to silver nanoparticles^33^. Prolonged exposure of AgNP may cause several developmental changes to the offspring through direct exposure or placental transfer^28,34–43^. Developmental exposure of rodent models to AgNPs has revealed changes in offspring organ development, such as in the fetal brain and mitochondrial disruptions leading to oxidative stress. However, detailed information on the developmental effects are still lacking and is an area of key interest^44^.

Extensive studies in zebrafish (*Danio rerio*), fruit flies (*Drosophila melanogaster*), and Mediterranean Sea urchins (*Arbacia lixula*) reveals that developmental exposure to AgNPs can lead to neurobehavioral alterations, gut-dysbiosis and other disturbances^45–54^. Select studies have also suggested that perinatal exposure to AgNPs can affect neurobehavioral programming and other offspring parameters in rodent models^36,43,55–57^, although more work is needed to understand the potential health effects in mammals of developmental exposure to such compounds.

AgNP can accumulate in the gastrointestinal (GI) tract, whereupon they can circulate to the brain. Recent findings support the notion that AgNPs accumulate in the brain after oral exposure, which indicates NPs might penetrate the blood–brain barrier (BBB) or be transmitted to the brain via retrograde axonal transmission^58^.

Besides targeting the host, silver and other NPs can affect the gut microbiome, although not all studies agree as to which bacteria if any are susceptible to such effects^33^. One study showed that rats orally exposed to AgNPs show decreased populations of *Firmicutes* and *Lactobacillus* but greater proportion of gram negative bacteria, which tend to be more pathogenic in the GI system^33^. Another study using next generation sequencing (NGS) technique demonstrated that oral exposure of AgNPs results in dose-dependent decreases in total gut microbiota composition and alterations in the proportion of specific bacterial species^59^. Examination of the effects of AgNPs on a defined bacterial community established from a healthy human donor reveals that these particles negatively influence the gut flora^60^.

In our previous study, we found that direct exposure of rats to AgNPs of different shapes such as cubes and spheres had an influence on the gut flora. Cube shaped AgNPs decreased *Clostridium* spp., *Bacteroides uniformis*, *Christensenellaceae*, and *Coprococcus eutactus* whereas, sphere shaped NPs reduced the relative concentrations of *Oscillospira* spp., *Dehalobacterium* spp., *Peptococcaeceae*, *Corynebacterium* spp., *Aggregatibacter pneumotropica*^61^. While it is clear that direct exposure to silver and other NPs can affect gut microbiota, it is not clear if AgNPs can exert effect on gut bacterial populations of mammalian offspring developmentally exposed to them and whether early exposure to such environmental chemicals can lead to long-term disturbances in the gut flora, otherwise considered gut dysbiosis. In *Drosophila melanogaster*, developmental exposure to copper or AgNPs, reduced progression to the larval and pupal stages, and larval climbing ability^48^. Correspondingly, developmental exposure to AgNPs reduced the overall diversity of gut microbiota relative to control larvae with those exposed to AgNPs containing increased relative amounts of *Lactobacillus brevis* but reduced amounts of *Acetobacter* relative to controls^48^.

It is important to understand how AgNPs and other environmental chemicals affect gut microorganisms as it is increasingly becoming apparent that shifts in gut bacteria can affect host health, especially neural development and correspondingly risk for neurobehavioral disorders, such as autism spectrum disorders (ASD). The intimate connection between the gut, microbiome, and brain has given rise to the term the microbiome-gut-brain axis^62–65^. The term implies that crosstalk exists between the gut microbiota and brain, with bacteria within the intestines able to modulate neurobehavioral responses, which could be due to direct effects of the bacteria, bacterial metabolites and other products that mimic host function, such as neurotransmitters, and/or stimulation of host inflammation.

The overarching hypothesis thus tested in the current study was that developmental exposure through the dam to AgNPs can affect neurobehavioral programming and the microbiome-gut-brain axis. To test this hypothesis, female mice were exposed to AgNPs or vehicle control 2 weeks prior to breeding and throughout gestation. Offspring were tested in various mazes that measure different behavioral domains, and the gut microbial profiles were surveyed from 30 through 120 days of age.

## Methods

Materials used in these studies, surface coating and dosage of AgNPs, sample preparation for silver quantification by ICP-OES, methods for Barnes maze, elevated plus maze (EPM), body composition analysis, brain histopathological analyses and morphometric quantification, collection of fecal samples, isolation of fecal microbial DNA, and 16s rRNA sequencing are included in Supplementary Material.

### Synthesis of silver nanoparticles (AgNPs)

AgNPs were synthesized based on a previously published procedure but with some modifications^66^. We sought to investigate the effects of pure monodispersed silver nanoparticles and therefore we utilized the sodium borohydride procedure. Briefly, sodium borohydride (10 mg; 26 mM) was dissolved in 10 ml distilled water and stored in dark at 4 °C for 30 min. Simultaneously, silver nitrate (42.4 mg; 2.5 mM) was dissolved in 100 ml deionized water in a 500 ml conical flask with vigorous stirring. Sodium citrate (40 mg; 3.1 mM) was dissolved in 100 ml DIW in 250 ml conical flask and added to silver nitrate solution and stirred vigorously for 5 min. Subsequently, 600 µl of sodium borohydride (2.5 mM) solution was added to the solution dropwise and stirred vigorously for 10 min. The color of the solution gradually changed to yellow. The temperature of the solution was then slowly increased to 100 °C and maintained for 90 min. At this point, the color of the solution was yellowish brown. The solution was cooled to room temperature and then kept in 4 °C overnight to allow the nanoparticles to grow. For the animal studies, three batches of 10X nanoparticle were prepared and pooled together. The pooled nanoparticle batch was passed through Minimate Tangential Flow Filtration (TFF) PALL filter (1 KD). The resulting concentrated, filtered solution was stored in 4 °C until further use. The pH of the nanoparticle solution was adjusted to pH 6.5 for animal studies.

### Animal husbandry and treatments

All animal studies were conducted according to University of Missouri approved Animal care and Use Committee protocols (protocol # 9590). The University of Missouri is accredited by the Association for Assessment and Accreditation of Laboratory Animal Care (AAALAC International). Mice were maintained under a 12:12 h light/dark cycle at 21 ± 1 °C and 50 ± 5% humidity. The Air Handler filter (W.W. Grainger Inc., Lake Forest, IL, USA) in the room was changed on a bimonthly basis. Standard laboratory diet (AIN93G phytoestrogen-free diet with 7% corn oil, Envigo, Madison, WI.The exact contents of the AIN93G phytoestrogen-free diet are listed in https://insights.envigo.com/hubfs/resources/data-sheets/94045.pdf. Diet and drinking water were available ad libitum. Twenty Female C57BL6 and 10 Male aged 4–5 weeks were purchased from Charles River and acclimatized for a period of 1 week. Female mice were randomly divided in to control and NP groups. Each group included 10 breeder females.

After 1-week of acclimatization, female mice in NP group received daily and oral administration of 3 mg/kg body weight (BW) of NP (200 µl, 0.4 mg/ml) via 18-gauge 75 mm long disposable gauge needles (Instech, Plymouth Meeting, PA). The control animals were dosed with same volume of sterile water (200 µl) as that of received by the NP group. After 1 week of receiving these treatments, females were paired with breeder males. Treatment with NP or water extended through gestation and ended at parturition. At 21 days of age, offspring were weaned. At this time, two male and two female offspring were randomly selected from each litter for follow-up gut microbiome and behavioral studies.

### Bioinformatics and amplicon analyses

Paired-end Illumina MiSeq DNA reads were joined using join_paired_ends.py and combined using add_qiime_labels.py from QIIME 1.9.1^67^. Uclust^68^ was used to clean contigs and remove those with E > 0.5, (http://drive5.com/usearch/manual/exp_errs.html). Contigs were clustered to 97% identity against DNA sequences in the Greengenes database^69^, version 13_8, using the QIIME^70^, version 1.9.1, script pick_open_reference_otus.py, which obviates chimera and PCR error detection with all reads clustered. After OTU selection, we filtered out OTUs with ≤ ten observation counts using the script filter_otus_from_otu_table.py.

Microbial diversity was evaluated by running alpha diversity and beta diversity on the OUT tables. Diversity analysis was conducted for each of the time points group (30, 60, 90 and 120 Days) using the q2-diversity plugin from QIIME2. The diversity comparisons were assessed between control and nanoparticles group. Firstly, biom format OTUs files were converted to QZA format used by QIIME2^71^ using “qiime tools import” command from QIIME2 package. For alpha diversity, Shannon’s diversity index (a quantitative measure of community richness), Observed OTUs (a qualitative measure of community richness) and Faith’s Phylogenetic Diversity (a qualitative measure of community richness that incorporates phylogenetic relationship between the features) values and rarefaction matrices were calculated and plotted using the “qiime diversity alpha-rarefaction” command supported by the QIIME2. Measurements of beta-diversity were facilitated by the QIIME2 command “qiime diversity core-metrics-phylogenetics” with PCoA plots generated. Visualization of taxonomy bar-charts were generated using “qiime taxa barplot” command in QIIME2.

LEfSe (Linear discriminant analysis effect size)^72^ was used to detect significant features. It combines the statistical test (Kruskal–Wallis test, pairwise Wilcoxon test) with linear discriminate analysis for feature selection and ranks feature by effect size. The alpha value employed for the factorial Kruskal–Wallis test and pairwise Wilcoxon test is 0.05. The threshold employed on the logarithmic LDA (Linear discriminant analysis) score for discriminative feature is 2.0.

### Microbial profiles temporal changes

In order to examine how the microbiome profiles changes over time, we co-assembled the abundant matrix for all samples. Average value of the significant taxa for each time group (30, 60, 90, and 120 days of age) was calculated to generate the circular plots using the anvi-interactive command in the Anvi’o^73^ tool. Each circular plot contains six layers. The auxiliary layers from the inside out include the bacterial genera and taxonomy name. The grey or black bars represent taxonomy abundance at 30, 60, 90, and 120 day of age.

### Functional metagenomics predictions

Bacterial metabolic characterization of sample types was facilitated with PICRUSt (the phylogenetic investigation of communities by reconstruction of unobserved states)^74^, version 1.1.4. We used the filter_otus_from_otu_table.py script from QIIME and Greengenes database (13_5_release) to discard any reads that did not hit the reference collections.

Normalize_by_copy_number.py script was used to normalize the OTU table by dividing each known/predicted 16S copy number abundance. The final metagenome functional predictions were predicted using predict_metagenomes.py script. We collapsed the results table, consisting of Kyoto Encyclopedia of Genes and Genomes (KEGG) Orthologs (KOs) at KO level 3 within the pathway hierarchy of KEGG using the categorize_by_functon.py script. DESeq2^75^ was used to highlight the KEGG terms that are significantly differentially abundant between control and NP samples with p < 0.1.

### Correlation of taxa abundance and metabolic activity abundance

To correlate the taxa abundance with metabolic characteristics of sample types, a custom R script provided as a gift from Dr. Jun Ma and Kjersti Aagaard-Tillery, Baylor College of Medicine, Houston, TX was used, as we have done previously^61,76,77^. We used similar methods for analysis as described in the previous report^61^. In these figures, the correlation of the abundance of taxa (from the OTU table) with the predicted metabolic function (from KEGG pathways as determined by PICRUSt), was calculated with the R stats function cor.test (https://cran.r-project.org/), using the Kendall method, a rank-based measure of association. The cor. test function outputs the correlation coefficient and significance of a comparison of an OTU with a KEGG term across samples. The matrix of all the correlation values was visualized using the R package corrplot (https://cran.r-project.org/). The area and intensity change together so that larger, darker, circles represent correlation coefficients that are larger in magnitude. The scale to the right of each figure relates those shades of color to the value of the correlation coefficient. Up to 53 of the most abundantly represented KEGG terms that had a significant (alpha < 0.05) correlation with one or more taxa were included in the plot^61^.

### Multi-omics integrative correlation analyses

We used the mixOmics R package^78^ to correlate the bacterial genera changes simultaneously with body composition, brain histological data and behavioral results, which enabled the integration of the microbiome, behavioral (Barnes, EPM), histology (Nissl and glial staining) and Echo results. We conducted sparse discriminant analysis with partial least square regression with function ‘block.splsda’. The circos plot was generated by using the ‘circosPlot’ function with correlations calculated using the method from González et al.^79^ and 0.7 correlation was used as the cutoff.

### Statistical analyses for histology and behavioral data

All dependent variables including social behaviors, Barnes maze, such as latency to find correct escape hole, and travel speed, EPM data, such as time spent in open/closed arms, head dipping and rearing, were analyzed as a split plot in space and time, as detailed in our previous studies^80,81^ and by using SAS version 9.4 program (SAS Institute, Cary, NC).

Barnes data was analyzed by using a repeated measurement design with PROC GLIMMIX. Barnes maze Latency data (as determined by the experimenter and the software program) were additionally analyzed by using a Proportional Hazard Ratio (PROC PHREG in SAS, Version 9.4, SAS Institute), as detailed previously. This analysis adjusts for right-censoring (defined here as not locating the escape hole within the allotted time) while still accommodating the study design. Effects are reported as a hazard ratio that represents the odds of a subject developmentally exposed to AgNPs finding the escape hole compared to individuals in the control group. A significant result indicates the odds are not 1:1. A result greater than 1 indicates the NP exposed group found the escape hole more quickly than the control group. A result smaller than 1 indicates that the NP exposed group found the escape hole more slowly compared to the control group. The litter was again used as the denominator of F for treatment, sex, day, and all possible interactions. These data are reported as the 95% lower confidence limit, mean, and 95% upper confidence limit. Treatment effects were tested with dam as the experimental unit and error term. Differences between treatment and control groups were determined by Fisher’s protected least-significant difference (LSD). The LSD was only calculated if the overall F test was significant^82,83^. A *p* value of ≤ 0.05 was considered significant. All data are presented as actual means (x̅) and standard error of the means (SEM).

## Results

### Synthesis and characterization of nanoparticles

AgNPs were synthesized following slight modification of reported procedure^66^. AgNPs were formed by the reduction of silver nitrate using sodium borohydride and sodium citrate as the stabilizer. The reagents were proportionally increased to prepare 10X concentration, and the final nanoparticle-solution was concentrated by PALL (1KD) filtration procedure. Dialysis was performed on concentrated silver nanoparticles to remove silver ions and the dialysates were used for the study^61^. The concentrated NP were characterized using Transmission electron microscopy (TEM), Dynamic Light Scattering (DLS) and UV–visible spectroscopy. TEM images confirmed the formation of highly dispersed and uniformly distributed spherical NP with an average core size of 18 ± 1.8 nm and characteristic surface plasmon resonance peak was observed at 400 nm (Figure S1). The hydrodynamic diameter (15.8 ± 3.4 nm) and negative zeta potential (− 32 ± 1.2 mV) are suggestive of highly stable and uniform particulate nature of the particles in solution. The increased absorbance of the NP confirmed the formation of NP at a higher concentration. Animals tolerated oral administration, and no observable changes were observed during the dosing periods. No morbidities or mortalities were observed in the female mice in the NP or control group.

### Barnes maze

As shown in Figure S2, latency to reach the escape hole and distance travelled was surprisingly reduced for mice developmentally exposed to NP compared to control group (Latency:115.31 ± 8.52 s and 126.19 ± 7.79 s respectively, *p* = 0.05; Distance Traveled: 7.34 ± 0.58 m and 8.42 ± 0.54 m, respectively, *p* = 0.01). However, there was no difference in velocity between the NP treated offspring and control groups. To further examine the differences that were evident for latency, the data were re-analyzed by using a Hazard Ratio analysis, which as detailed in the methods section, are controls for those animals that did not find the maze in the allotted 300 s. This approach was also used to analyze the results per each day of the 5-day trial period (Figure S3). AgNPs exposed offspring were more likely to find the escape hole than controls on Day 4 (1.81; *p* = 0.04), and this trend continued through Day 5 (1.38; *p* = 0.06).

### Elevated plus maze (EPM)

**Figure 1:**
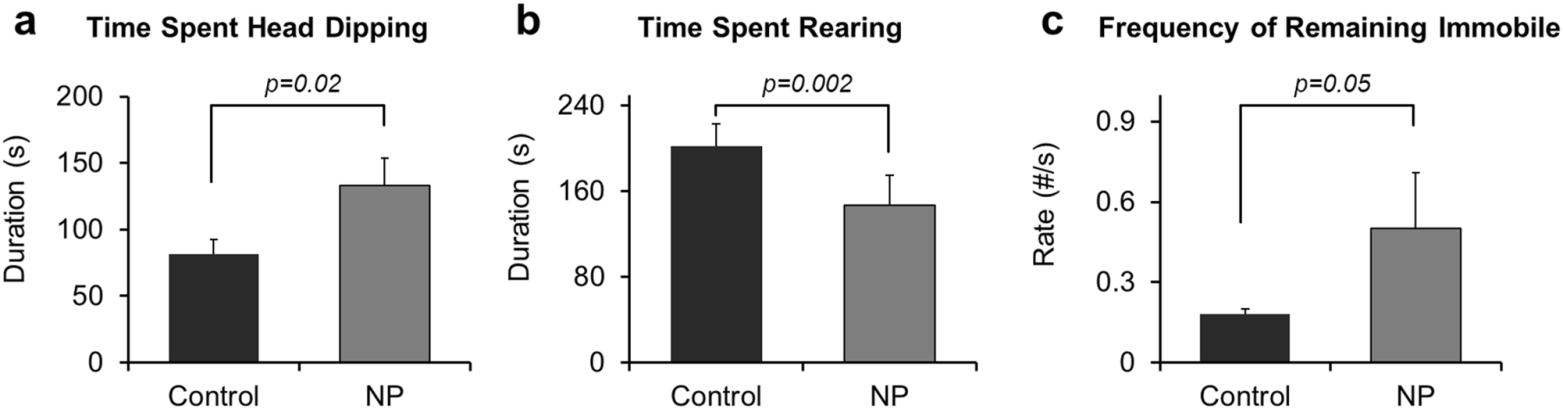
EPM results: (**a**) Time spent head dipping, (**b**) Time spent rearing and, (**c**) Frequency of remaining immobile. *p* value differences are designated above the graphs. Replicates tested include n = 11 for Ctrl; n = 12 for NP (AgNP).

Mice developmentally exposed to NP spent more time engaged in stereotypic head dipping behaviors relative to controls (132.75 ± 21.11 s and 81.65 ± 11.08 s, respectively; *p* = 0.02, Fig. 1a). Rearing is considered another stereotypical behavior. However, mice developmentally exposed to NP, had lower frequency of rearing (0.75 ± 0.13 n/s), compared to controls (1.39 ± 0.27 #/s, *p* = 0.05) and thus, the average time spent rearing by mice treated with NP (146.81± 27.68 s, Fig. 1b) was reduced compared to controls (201.71 ± 21.02 s; *p* = 0.002). The incidences of remaining immobile during the testing period were lower in NP treated offspring (0.50 ± 0.21 n/s) relative to controls (0.18 ± 0.02 #/s; *p* < 0.05) (Fig. 1c). None of the other parameters, including number and duration of time spent in the different arms or center differed between the two groups (Table S2).

### Histopathology

**Figure 2:**
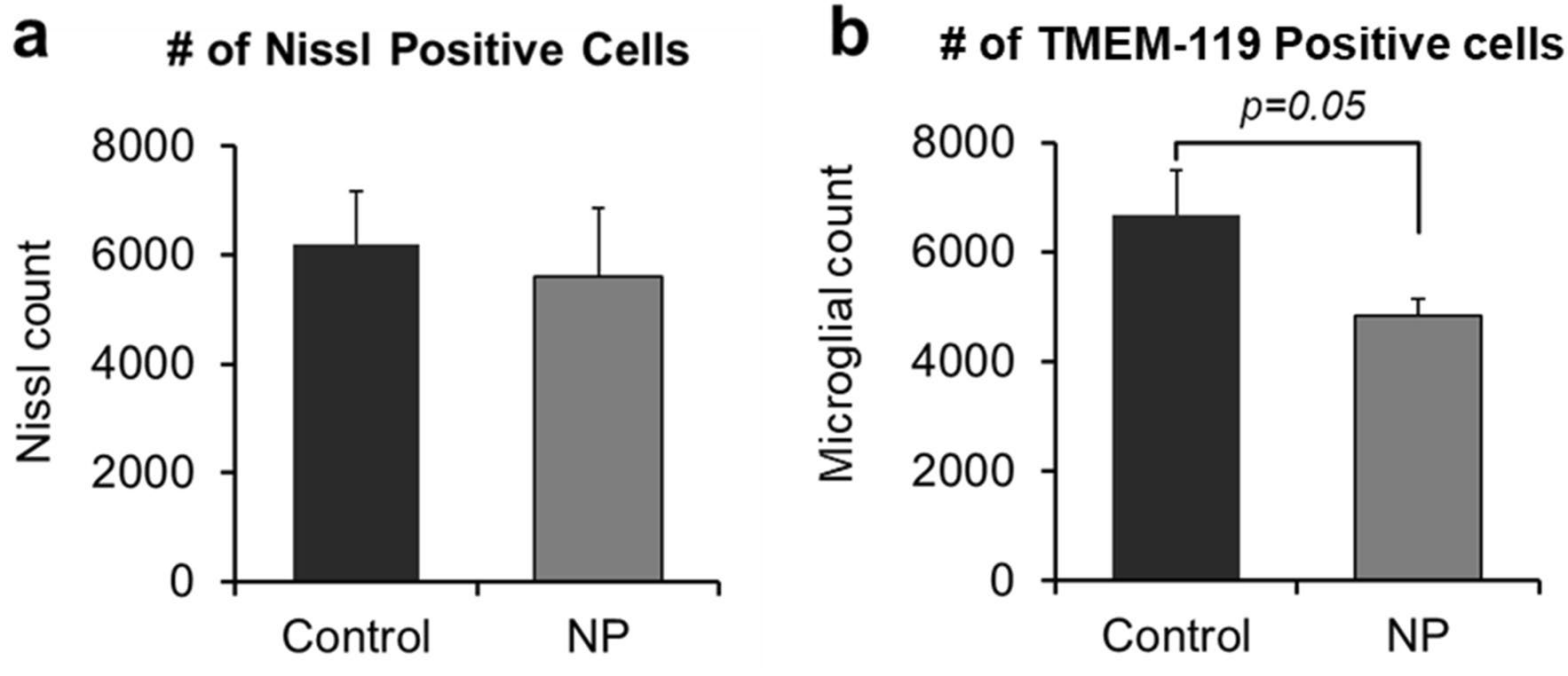
Nissl and Glial staining results: (**a**) Nissl positive cells and (**b**) Microglial positive cells. *p* value differences are designated above the graphs. Replicates tested include; n = 11 for Ctrl; n = 12 for NP (AgNP).

Neuronal cell counts, as determined by Nissl staining, for the NP treated (6183.75 ± 1000.96) and control group offspring (5613.73 ± 1245.27; Fig. 2a) were similar. However, the microglial counts were reduced in the NP treated offspring (4838.27 ± 293.96) compared to the controls (6681.92 ± 824.31, *p* = 0.05, Fig. 2b). Brain sections containing hippocampus stained with Nissl dye for neurons and *anti*-TMEM for microglial cells are shown in Figure S4.

### Echo MRI

**Figure 3:**
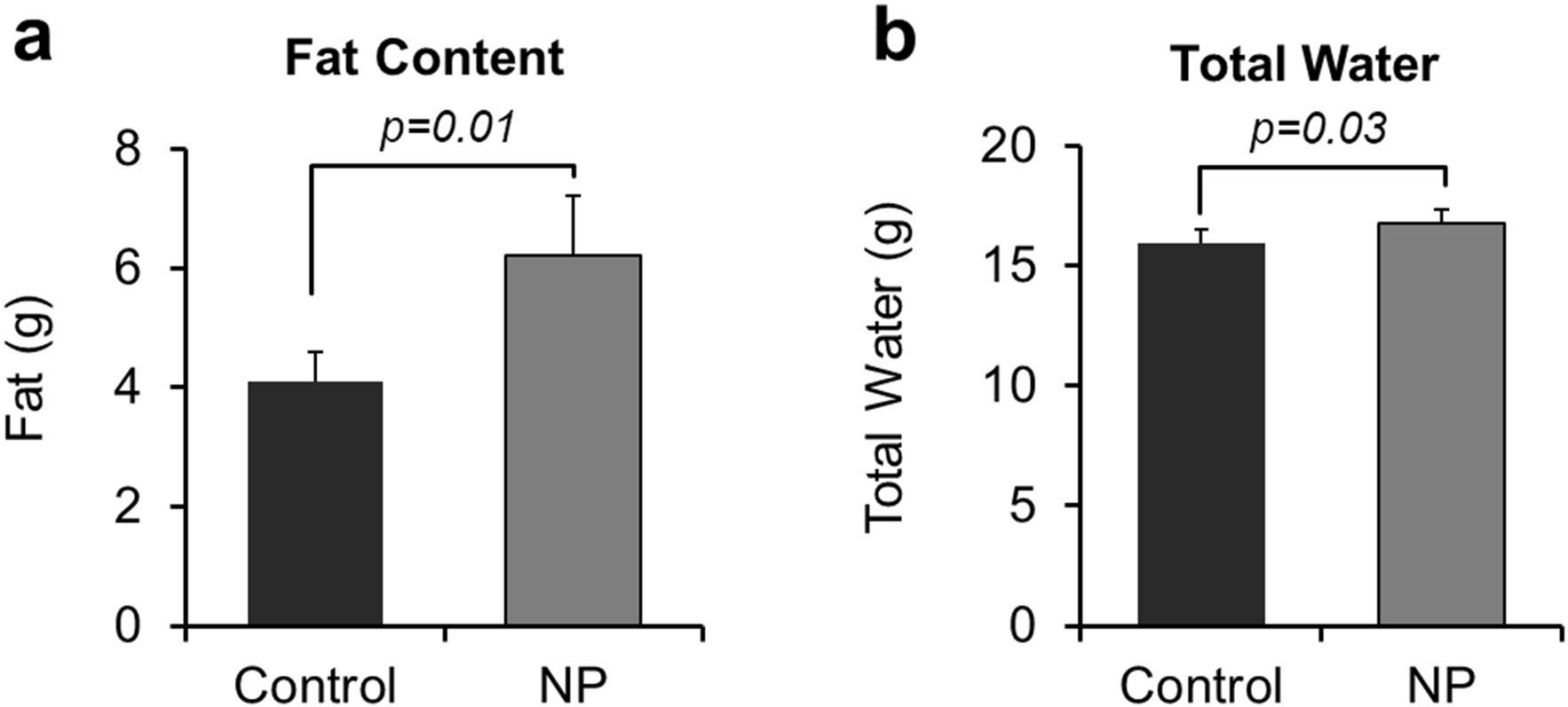
Echo-MRI results for: (**a**) Total fat content and (**b**) Total water. *p* value differences are designated above the graphs. Replicates tested include n = 11 for Ctrl; n = 12 for NP (AgNP).

Mice developmentally exposed to NP had increased fat content (6.20 ± 1.01g) relative to controls (4.11 ± 0.47 g; *p* = 0.01, Fig. 3a). Total water content for mice developmentally exposed to NP (16.76 ± 0.60 g) was slightly higher than control group (15.96 ± 0.56 g; *p* = 0.03) as shown in Fig. 3b. No significant differences were observed in total lean mass, lean percentage, free water, free water percentage and total water percentage (Table S3).

### Gut microbiota

**Figure 4:**
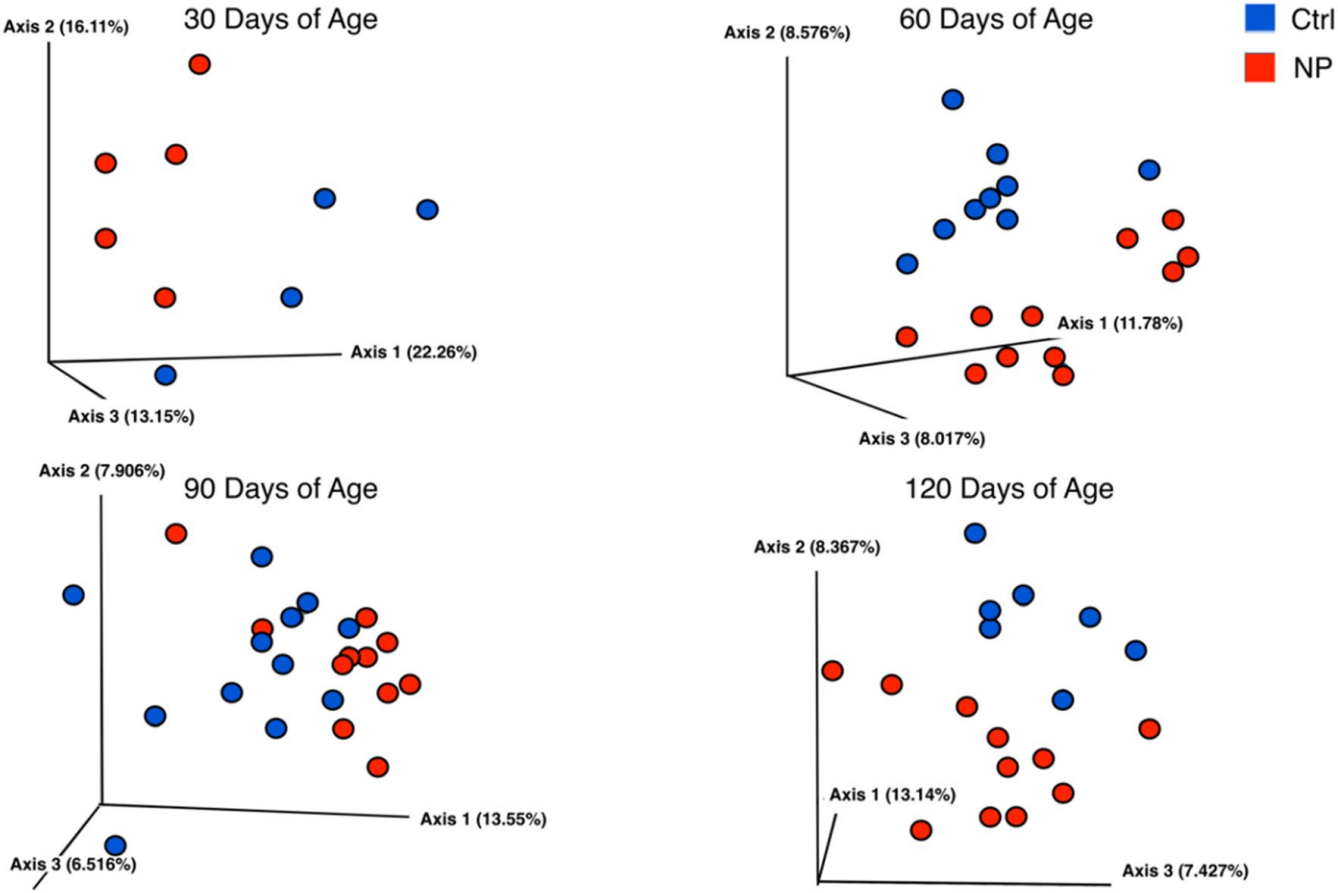
3D PCA plots to show β-diversity. Figure shows results for Control and NP (AgNP) groups at 30, 60, 90, and 120 days of age. PERMANOVA values: Replicates tested include Ctrl and NP (AgNP) groups respectively at 30 (n = 10 and n = 9), 60 (n = 11 and n = 11), 90 (n = 12 and n = 10), and 120 (n = 10 and n = 12) days of age. 30 days of age *p* = 0.027, 60 days of age *p* = 0.001, 90 days of age *p* = 0.002, and 120 days of age *p* = 0.006. Each circle represents a single replicate.

We first considered whether developmental exposure to AgNPs altered the α- and β-diversity at 30, 60, 90, and 120 days of age. Shannon analysis revealed no differences based on treatment at any of these ages in α-diversity (Figure S5). However, 3D PCA analyses reveal clear separation based on treatment group with the PERMNANOVA values ranging on these days from *p* = 0.001 to 0.03 (Fig. 4).

**Figure 5:**
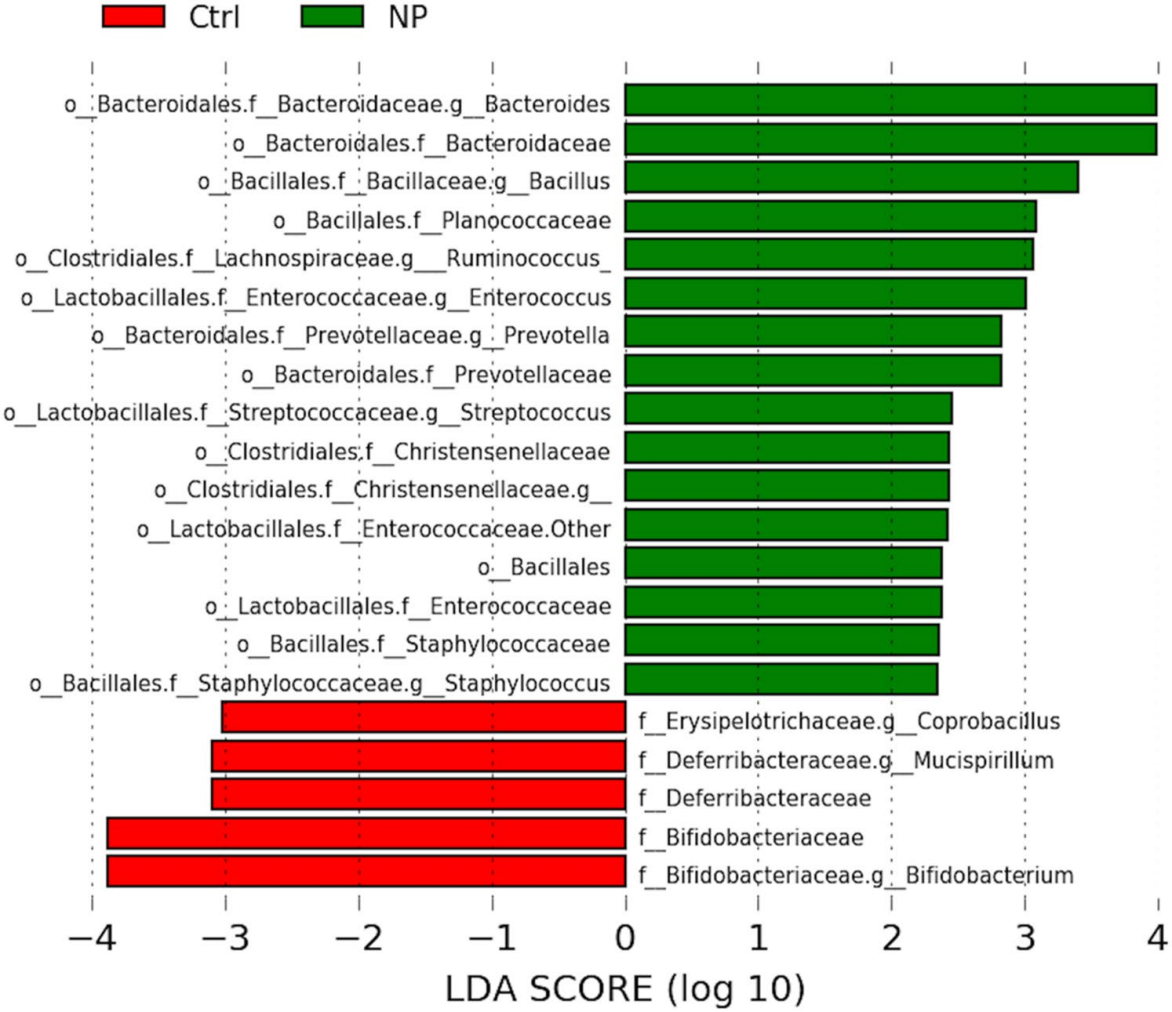
LEfSe analyses for bacterial differences at 120 days of age. Bacteria shown in green bars are greater in NP (AgNP) group; whereas those shown in red are greater in control (Ctrl) group. Replicates tested include n = 10 for Ctrl; n = 12 for NP (AgNP).

As the behavioral studies were conducted between 90 and 120 days of age, we further pursued those bacteria that differed at 120 days of age by analyzing the data with LEfSe analyses. As shown in Fig. 5, developmental exposure to AgNPs resulted in the relative expression of several bacteria increasing, although there were select ones that were downregulated due to this exposure. Examples of bacteria that were upregulated in the NP group include *Bacteroides* spp., *Bacillus* spp., *Ruminococcus* spp., *Planococcaceae*, *Enterococcus* spp., *Prevotella* spp., *Staphylococcus* spp., *Streptococcus* spp., *Christensenellaceae*, and *Enterococcaceae.* In contrast, *Coprobacillus* spp., *Mucispirillum* spp., and *Bifidobacterium* spp., were reduced in those developmentally exposed to NP.

In order to examine how the microbiome profiles change over time, circular plots using the Anvi’o program^73^ were generated. Each circular plot contains six layers. The auxiliary layers from the inside out include the bacterial genera and specific taxonomy name. The grey or black bars represent taxonomy abundance at 30, 60, 90, and 120 day of age. As shown by increasing amount of black, *Bifidobacterium* spp. is moderately present in controls at 30, 60, and 90 days of age, and then dramatically increases at 120 days of age (Figure S6). However, this bacterium is less abundant in those developmentally exposed to NP at all ages examined. *Bacteroides* spp. is plentiful in control and NP at all ages examined. The relative abundance of *Prevotella* spp. is greatest at 30 days of age in controls and NP and decreases thereafter. *Mucispirallum* spp. relative abundance is most plentiful in controls at 30 and 120 days of age; whereas, in NP, this bacterium peaks at 60 days age. In controls, *Ruminococcus* spp. is abundant at 30 days of age and declines thereafter. In contrast, this bacterium is abundant at 60 days of age in NP group and declines but still present at 90 and 120 days of age.

**Figure 6:**
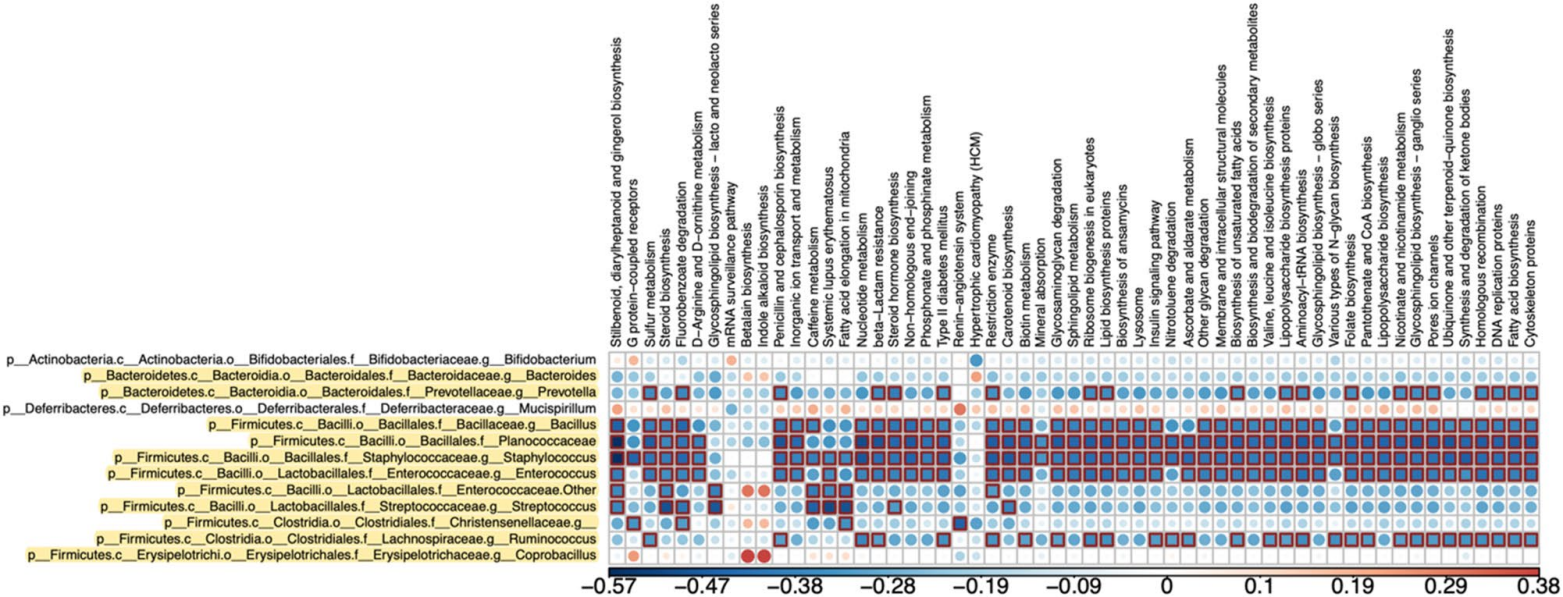
Bacterial metabolic and other pathway differences in the fecal microbiome of NP vs. Ctrl. As described in Fig. 7 of Ma et al.^103^, correlations between the PICRUS t-generated functional profile and QIIME-generated genus level bacterial abundance were calculated and plotted against treatment group. Those genera that were identified by LEfSe as being different between the two groups are depicted. Bacteria that are highlighted had increased relative amounts in NP (AgNP) group. Metabolic pathway designations are delineated at the top of the figure. Shading intensity and size of the circles indicates the Kendall rank correlation coefficient between matrices. Red indicates a positive correlation; whereas blue designates a negative correlation. Red squares surrounding the circles are indicative of a P value ≤ 0.05. Legend for the quantitative scores associated with the range of blue to red colors is listed below the figure.

Correlation of PICRUSt analysis with bacterial changes reveals that the most significant metabolite associations are with those bacteria upregulated in the NP, including *Prevotella* spp., *Bacillus* spp., *Planococcaceae*, *Staphylococcus* spp., *Enterococcus* spp., and *Ruminococcus* spp. (Fig. 6). For *Prevotella* spp., example KEGG pathways and metabolites that were inversely associated with it include sulfur metabolism, flurobenzoate degradation, penicillin and cephalosporin biosynthesis, beta-lactam resistance, steroid hormone biosynthesis, type II diabetes, lipid biosynthesis proteins, biosynthesis of unsaturated fatty acids, aminoacyl t-RNA synthesis, and folate synthesis. All of these were also inversely associated with *Bacillus* spp., *Planococcaceae*, *Staphylococcus* spp., and *Enterococcus* spp. Additional ones that were negatively associated with these bacteria include steroid biosynthesis, D-arginine and D-ornithine biosynthesis, nucleotide metabolism, biotin metabolism, sphingolipid metabolism, lysosome, other glycan degradation, valine, leucine, and isoleucine biosynthesis, glycosphingolipid metabolism, pantothenate CoA biosynthesis, and lipopolysaccharide biosynthesis. Example pathways and metabolites inversely associated with *Ruminococcus* spp. include penicillin and cephalosporin biosynthesis, nucleotide metabolism, beta-lactam resistance, biotin metabolism, biosynthesis of unsaturated fatty acids, aminoacyl t-RNA synthesis, and folate synthesis. No associations were observed for those bacteria down-regulated in the NP group.

### Integrative correlation analyses

**Figure 7:**
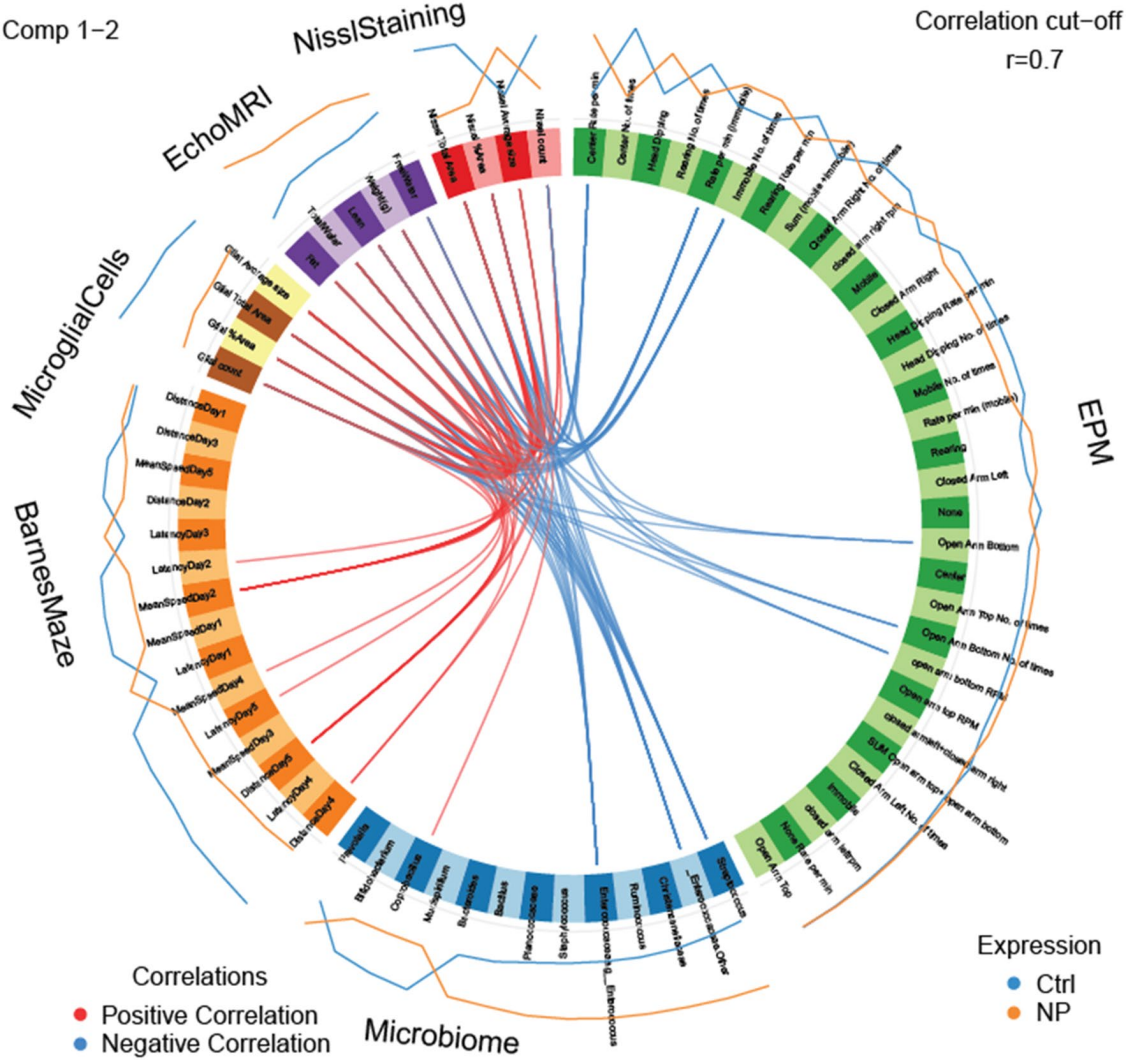
Circos plot correlations between gut bacterial, brain histological analyses, and behavioral parameters in NP vs. Ctrl. Red lines in the center indicate a positive correlation. In contrast, blue lines indicate a negative correlation. Results for AIN females are indicated with a blue line outside of the circle. Orange line indicates results for GEN females. The color of the line further from the circle indicates the group where these results are greater.

We used mixOmics analyses^78^ to correlate the neurobehavioral and metabolic phenotyping and gut microbiota results with the correlation value set to r = 0.7, as recommended. As shown in Fig. 7, *Coprobacillus* spp., which was reduced in the NP group, positively correlated with number of Nissl positive cells. *Enterococcus* spp. and *Enterococcaceae*, both of whose relative expression were increased in NP exposed individuals, inversely correlated with body weight, fat content, and glial cell numbers. Streptococcus, which was increased in the NP group, negatively correlated with body weight, fat content, Nissl, and glial cell numbers.

## Discussion

The goals of the present study were to determine if developmental exposure of dams to AgNPs at environmentally relevant concentrations affect the microbiome-gut-brain axis and causes long-term neurobehavioral changes in mouse offspring. Further, histopathological examination of hippocampus was performed to examine the potential changes in neuronal and microglial cells due to maternal AgNPs exposure.

Citrate coated spherical AgNPs are utilized for several applications. However, the developmental effects have not been clearly studied, and therefore, we examined these NPs for our present investigation^36^. The gastrointestinal (GI) fluid is acidic with a pH ranging from 3–7 depending upon the presence of digesta within. The fate and the bioavailability of the ingested particles are critically influenced by these GI tract fluids upon oral ingestion. Several groups have conducted studies to understand the integrity of AgNPs in biological fluids^84–89^. These studies suggest that the physiological conditions, such as presence of digesta, pH, and ionic concentrations, of the gastrointestinal tract affects NP size and coating and other various^87–93^. Only a handful of studies have been conducted to perform detailed investigation on the exposure effect of gastrointestinal fluids. Findings reported on citrate stabilized AgNPs (40 nm) to synthetic human stomach fluid (pH 1.5) suggest that these partially react to form aggregates along with silver chloride. However, this study may not be representative of AgNP preparations in general^87^. We have examined exposure of AgNPs coated with polyvinyl pyrrolidone to GI fluids (at three pH concentrations 3, 7 and 9) and subsequent effects on the zeta potential and hydrodynamic diameter^61^. Our data show aggregation of NPs in the lower pH range, but notably, the particulate nature is maintained. Taken together, it is clear that bioavailability and accumulation of AgNPs can vary based on GI state, but, the aggregate nature of such compounds persists.

Developmental disturbances may originate due to direct and indirect effects of nanoparticle exposure. Studies show that AgNPs can be transferred from dam to offspring through the placenta and milk^25^, whereupon they can permeate through the blood brain barrier^26^. The effects of developmental exposure to AgNPs in mammals is not completely understood AgNPs may persist for up to at least 4 months in the dam; whereupon, they have the potential to cross the placental barrier and accumulate in the fetus^34,39^. For instance, one study that used an oral exposure approach with AgNPs in dams, who were exposed prior mating and during gestation, showed accumulation of these particles in brain and other organs of her 4-day old offspring^37^. A short-term study using radioactive NPs, has confirmed the transport of ~ 50 nm AgNPs (polyvinyl pyrrolidine coating) that maternal oral exposure leads to placental and lactational transfer and subsequent accumulation in the neonatal pup brains^35^. While there have been select studies, as detailed below, examining how such direct exposure to AgNPs might affect later neurobehavioral domains, we sought to do a comprehensive assessment of such effects.

In the first set of studies, we examined whether developmental exposure to AgNPs would affect various neurobehavioral domains and metabolic parameters. Spatial navigation and learning were tested with the dry land Barnes maze. The overall trial results, however, are inconclusive, as on select trial days those exposed to AgNPs surprisingly showed some improvement over controls, but this effect was only marginal. Distance travelled was shorter for those developmentally exposed to NP, while there was no change in the velocity between the two groups. Further behavioral tests, such as the Morris Water Maze (MWM) or Y maze, are needed to validate the current findings and determine whether exposure to AgNPs affect spatial learning and memory. We opted at the outset to use the Barnes maze as it is considered more ecologically relevant for terrestrial rodents^94^.

In another developmental study, spatial learning and memory, as determined by the MWM, and hippocampal neuro-development appear to be altered in a dose dependent manner in offspring prenatally exposed to citrate coated AgNPs. Impaired cognition was observed at a dose of 2 mg/kg BW subcutaneous administration^34^. One potential explanation for differences observed in our study relative to this one is the route of administration. We used oral route of administration to mimic dietary exposure, whereas this aforementioned study subcutaneously injected the dams starting from gestational day 3 with repeat administration every 3 days up until parturition.

Anxiety-like, exploratory, and repetitive behaviors, as measured with the EPM, were also evaluated in the same groups of offspring. Head-dipping and rearing behaviors are considered repetitive behaviors. The results of our study indicate that mice developmentally exposed to NPs spent less time in head-dipping incidences compared to controls. Yet, this latter group also showed reduced duration of rearing, which is also considered as a stereotypical behavior. The incidences of NP treated offspring being immobile were greater to controls. Taken together, our findings suggest that the mice exposed to AgNPs might be more anxious and more likely to engage in at least one stereotypical behavior, head dipping. Follow-up studies, such as light–dark box (LDB) tests are required to validate these initial behavioral observations.

No changes in anxiety-like behavior was observed in mice offspring as assessed by EPM and LDB test following subcutaneous exposure of NPs in prenatal stage^34^ and oral exposure during lactation stage^38^; however, developmental exposure led to neural toxicity in mouse fetuses, as evidenced by mitochondrial autophagy, dysmorphology, and dysfunction of neuronal cells, blood brain barrier inflammation and astrocytic swelling^95^. Another study with mice suggests that prenatal exposure to AgNPs (2 mg/kg) results in increased time female but not male offspring spend immobile when tested in the forced swim test^56^. The finding suggests that developmental exposure to AgNPs increases depressive-like behaviors in female offspring. Dopamine is a key neurotransmitter regulating emotive behaviors, and rat offspring exposed through the dam to AgNPs have increased expression of genes involved in dopamine metabolism, including tyrosine hydroxylase (*Th*) and monoamine oxidase A (*Maoa*)^57^.

Histological examination was performed in hippocampus region of the brain to correlate the differences in spatial, memory, behavioral and cognitive learning. There were no significant differences in the neuronal counts between the offspring developmentally exposed to NPs and the control groups, indicating that NP exposure had minimal effect on the cognitive behavior. Microglial cells are the macrophages in central nervous system and most likely to respond when NPs enter the brain. Lower microglial counts (resident macrophages in the brain) suggest AgNPs exposed offspring might be more vulnerable to pathogens. There are limited in vivo studies detailing how direct or developmental exposure to AgNPs might affect microglial cells in the brain. However, a review on central nervous system toxicity suggests that nanomaterials can affect microglial function^96^.

In this study, we used echo-MRI to assess how developmental exposure to AgNPs affect body condition. Echo-MRI indicated increased body fat content in NP exposed offspring. The increased body fat content suggests the possibility of metabolic disorders, including fatty liver disease Such type of disorders has been reported due to oral exposure of AgNPs in overweight mice^97^. However, additional work is needed to characterize the full extent of metabolic disruptions due to developmental exposure to AgNPs.

One of the other goals of the current set of studies was to determine whether developmental exposure to NPs could affect gut microbiota populations from weaning to adulthood in mice. Our past studies with rats examined the effects of NPs on gut microbiota focused solely on direct effects^30,45–54,59,60,98^. However, to the best of our knowledge, no studies have examined whether *in utero* exposure to NPs might deleteriously affect the initial bacterial colonization of the gut that would lead to permanent changes on composition of the gut flora. This issue is of critical importance given the fact that the gut microbiome can lead to profound effects on the host, including increasing the risk for neurobehavioral and metabolic disorders^61,62,99^.

The current studies clearly demonstrate that developmental exposure to AgNPs induces long-term effects on gut microbiota with the greatest divergence from control mice occurring at the last timepoint assessed, 120 days of age. As shown though in the 3D PCA diagrams, at all ages tested gut bacteria within those exposed early on to AgNPs clustered separately from controls. Since, 120 days of age led to the greatest separation between these two groups and this was also around the period of the behavioral and metabolic testing, we focused on those bacteria and associated predicted metabolic pathways that differed at this timepoint. The prediction at the outset was that the antimicrobial properties of AgNPs would lead to a general reduction in most bacteria. However, the relative abundance of several bacteria was greater in the AgNP exposed group relative to controls. These include *Prevotella* spp., *Bacillus* spp., *Planococcaceae*, *Staphylococcus* spp., *Enterococcus* spp., and *Ruminococcus* spp. The few bacteria that were reduced in those developmentally exposed to AgNPs were *Coprobacillus* spp., *Mucispirillum* spp., and *Bifidobacterium* spp.

An increased in the relative abundance of *Prevotella* spp. in humans is associated with greater body mass index (BMI)^100^. This bacterium is also linked with colitis and enhancement of intestinal inflammation^101^. An over-abundance of a *Ruminococcus* spp. occurs in human patients with generalized anxiety disorder^102^ and in children with Pervasive Developmental Disorder Not Otherwise Specified (PDD-NOS)^103^.

Of the bacteria who relative abundance is decreased in those developmentally exposed to NP, the most concerning is reductions in *Bifidobacterium* spp. Several health benefits are associated with this bacterium, and it is thus included in many probiotic treatments. One study showed that supplementation of a probiotic containing *Bifidobacterium* spp. led to improved health outcomes in children with ASD and gastrointestinal disorders^104^. Reductions in this bacterium may render rodent models and humans susceptible to neurobehavioral and GI diseases. If so, it would be of interest to determine whether supplementation of *Bifidobacterium* to mice developmentally exposed to NP alleviated some of their clinical signs.

PICRUSt analyses showed that several pathways were negatively associated with bacteria up-regulated in those developmentally exposed to NPs. This association means that as these bacteria increased in the NP group, such pathways are predicted to decrease. Amongst these pathways of importance is the inverse association of *Prevotella* spp. and steroid hormone biosynthesis. Steroid hormones, including testosterone and aromatization of testosterone to estrogens, is essential in regulating many key neural functions^105–108^. Reductions in these steroid hormones, especially estrogens, is linked to later neurobehavioral disorders, such as Alzheimer’s Disease^105,106^.

Biosynthesis of unsaturated fatty acids is another pathway likely reduced in response to increases in *Prevotella* spp. and *Ruminococcus* spp. Unsaturated fatty acids, in particular docosahexaenoic acid (DHA), are essential for normal cognitive functions including learning and memory, coping with stress, regulating other emotional response, inhibiting inflammation in the brain, reducing the risk of impulsive disorders, AD, and other brain disorders^109–111^. The findings thus suggest that NP-induced changes, including enrichment of select bacteria, may correspondingly affect metabolic pathways. Changes in such pathways may serve as one mechanism linking gut microbiome to neurobehavioral alterations.

For integrative correlation analyses with the mixOmics program, the potentially most meaningful correlation was the inverse relationship between microglial cell numbers, which were reduced in the NP group, and *Enterococcus* spp., *Enterococcaceae*, and *Streptococcus* spp., all three bacteria that were increased in the NP group. In general, bacterial changes are associated with increase host inflammation^62^. Thus, the linkage between these bacteria and reductions in microglial cells, resident macrophages in the brain, are surprising. The findings should be taken with caution as they are only correlative. The reductions in microglial cells could also be due to effects of developmental exposure to AgNPs as shown in other studies that examined direct effects in adult rodent models^112^.

The major goal of the study was to investigate the long-term consequences of early in utero exposure, otherwise considered as developmental origins of health and disease (DOHaD) changes. This concept suggests that for better or worse effects can be observed long after the exposure to a factor, in this case AgNPs, has ceased. As such, quantifiable amount of AgNPs in adult offspring organs, such as the gut and brain, would presumably be below the detection limits of traditional analytical procedures. The more germane concern is whether such materials can be transferred via the placenta and possibly the milk during the perinatal period. Indeed, previous reports indicate that AgNPs can be transferred from dam to offspring through the placenta and breast milk 25, 26 in rodents and may lead to developmental disorders 28, 34-43.

## Conclusions

In the present study, we investigated the neurobehavioral and gut microbiota changes due to developmental exposure to AgNPs. Our results indicate that developmental exposure of AgNPs to mice can lead to increase likelihood of engaging in certain repetitive behaviors. AgNP exposure resulted in reduction of resident microglial cells, that may render exposed offspring more vulnerable to infection. Such effects on the brain and behavioral domains might be either direct in origin or due to AgNP-induced changes on the gut microbiome. Future studies with germ-free mice will be useful in teasing apart these two potential mechanisms. The current studies though raise cause for concern that developmental exposure to AgNPs can lead to longstanding effects on the microbiome-gut-brain axis.

## Supplementary Information

Supplementary Information.
