# Supplementary material for: Developmental exposure to silver nanoparticles leads to long term gut dysbiosis and neurobehavioral alterations
Source: Sci Rep. 2021 Mar 22;11:6558. doi: 10.1038/s41598-021-85919-7 (PMC7985313; doi:10.1038/s41598-021-85919-7)
Supplement: Supplementary file 1 — Supplementary Information. [file 41598_2021_85919_MOESM1_ESM.docx]

SUPPLEMENTARY

**Developmental Exposure to Silver Nanoparticles Leads to Long Term Gut Dysbiosis and Neurobehavioral Alterations.**

Author information

Zhen Lyu^Ψ,1^, Shreya Ghoshdastidar^Ψ,2^, Karamkolly R. RekhaΨ^,3^, Dhananjay Suresh^2^, Jiude Mao^4,7^, Nathan Bivens^5,^ Raghuraman Kannan^2,3^, Trupti Joshi^1,6,7,8★^, Cheryl S. Rosenfeld^4,7,8,9,10,11★^, Anandhi Upendran^11,12★^

^Ψ^ Equal contributing authors

Author affiliation

Departments of ^1^Electrical Engineering and Computer Science, ^2^Bioengineering, ^3^Radiology, ^4^Biomedical Sciences, ^5^DNA Core Facility, ^6^Health Management and Informatics, ^7^MU Institute of Data Science and Informatics, ^8^Bond Life Sciences Center, ^9^Genetics Area Program, ^10^Thompson Center for Autism and Neurobehavioral Disorders, ^11^Medical Pharmacology & Physiology and ^12^MU-Institute of Clinical and Translational Science, University of Missouri, Columbia, Missouri 65212, USA

^★^Correspondence: [UpendranA@health.missouri.edu](mailto:UpendranA@health.missouri.edu); [RosenfeldC@missouri.edu](mailto:RosenfeldC@missouri.edu)[;JoshiTr@health.missouri.edu](mailto:RosenfeldC@missouri.edu;%20JoshiTr@health.missouri.edu)

***Materials and Methods***

*Materials*

Silver nitrate and Sodium citrate dihydrate were purchased from Acros Organics. Sodium Borohydride was purchased from Sigma Aldrich. Ultrapure distilled water was purchased from Invitrogen. Ultra-filter membranes (1KD Minimate TFF) were purchased from PALL Corporation (NY, US). Cresyl violet solution was procured from thermo fisher scientific. Antibodies were procured for IHC were procured from Abcam (Cambridge, MA, US) Phosphate buffer solution, BSA and other reagents were procured from Sigma, Millipore (St. Louis, MO, US). TEM images were recorded to measure the core size, while the hydrodynamic size and zeta potential of the measurements were performed using Malvern Zetasizer (USA). The amount of silver was quantified using Varian Vista-MPX CCD Simultaneous Inductively coupled Plasma – Optical Emission Spectroscopy (ICP-OES) (US).

*Sample preparation for silver analysis and calibration for ICP-OES analysis:*

Microwave digestion was used for sample preparation. 10 μL of concentrated HNO_3_ was added to the sample vial. A preloaded method for the MARS (CEM, corporation, USA) microwave was used to digest the sample. Triplicate measurements were performed to measure the silver concentration. Once the sample was completely digested and cooled, the digest was diluted with DI water up to 50 ml volume and mixed well. 5 μg/mL silver standard (Assurance LPC standard 1; SPEC Ceri Prep) used for ICP-OES analysis was used for calibration and the amount of silver in the digested solution was quantified. The total amount of silver present in the dialysates as quantified by ICP-OES was 468 *ppm*.

*Surface coating and dosage of silver NPs*

Citrate coated spherical silver NPs are most commonly used in a wide array of applications, and thus of greatest human health concern.^1^ Our previous short-term low dose exposure study suggested that spherical particles are prone to cause changes compared to cube shaped particles.^2^ Therefore, in the current studies we tested citrate coated spherical silver NPs of size range 20 nm for the present study. A dose of silver NPs chosen for the present study was 3 mg/Kg BW, in pre and postnatal development of mice. This dose is within the NOAEL (No observed adverse effect level) of silver NPs as tested in developmental studies. It is also well within the toxicity range proposed for possible human and rodent silver exposure.^3-12^ Similar oral exposure studies in rodents have been reported to result in accumulation of particles in a variety of organs, including the brain, and developmental changes.^3-8, 10, 12-15^

*Offspring Behavioral Studies*

*Barnes maze Test*

At 90 days of age, Barnes maze testing, which measures spatial learning and memory, was performed on offspring as described previous.^16-18^ The Barnes Maze circular platform includes 12 evenly spaced holes placed along the perimeter. An enclosed tunnel was placed under one escape hole and all the other holes remained closed. The escape hole number for each mouse was assigned by using a random number generator. The assigned hole number was used for each mouse for all the five days of the trial. The experiments were conducted at the same time (13:00 to 15:00 hours) on all the five days. The mice were placed in the center of the maze and given up to 600 seconds to find the escape hole. To encourage the mice to locate the escape hole, bright lights were placed above the maze. Stimulatory light measured ≈1,200 lx (vivarium room lighting measured 420 lx).^19^ On the first day if they did not find it, they were gently guided to it. A stopwatch (Fisher Scientific, St. Louis, MO) was used to ensure that the trial only lasted up to 600 seconds. Upon entering the escape hole, the stopwatch was immediately stopped and trial ended. The maze was cleaned between trials with 70% ethanol. This also removed odor cues of the mice. The activity of the mouse within the maze was recorded by using a Canon Vixia HF HD (Cannon, US) video camera. The mouse was permitted to rest for ½ hour, and a second trial was then performed. From the videos, distance covered (m), duration (latency, s) and speed (m/s) were determined by using the Any Maze software (Wood Dale, IL, US).

*Elevated Plus Maze (EPM) Testing*

Elevated plus maze (EPM) test was used to assess anxiogenic, exploratory, and repetitive behaviors in the mice, as detailed previously.^2, 20, 21^ Briefly, the EPM is arranged in a plus configuration and includes two opposite open arms (30 cm), a central platform region (5 × 5 cm), and two opposite closed arms (30 cm). The maze was supported 100 cm above the floor by a stand constructed of polypropylene.^19^ Each animal was placed in the center of the maze and permitted to explore it for 300 seconds. After each test, the apparatus was cleaned with 70% EtOH. Each trial was recorded with a Canon Vixia HF HD hand held camcorder (Canon, US). The video trials were then analyzed with the Observer Version 11 software (Noldus Technologies, Leesburg, VA). Parameters measured include duration of time spent and frequency entering the open and closed arms, center of the maze, duration and number of times engaged in head-dipping and rearing behaviors. The trial period was measured with a stopwatch (Fisher Scientific, St. Louis, MO).

*Body composition analysis*

The body composition, including total fat content, free water, total water and lean mass were measured using magnetic resonance imaging (EchoMRI-1100, EchoMRI LLC, Houston, TX, USA) following standard protocols ^16^. Echo-MRI was performed at the end of behavioral study prior to euthanasia, and the data analyzed as reported previously ^22^.

*Histopathological analyses and morphometric quantification*

At the end of behavioral studies, mice were humanely euthanized in accordance with AVMA Guidelines for Laboratory Animal Euthanasia. Following euthanasia, mice brains were quickly isolated and the whole brain was horizontally dissected and fixed with 4% paraformaldehyde (PFA) solution. After PFA fixation, brains were transferred to sucrose solution (10-30%). Samples transferred to 30% sucrose were kept at 4°C for 24 hours, following which they were placed in pre-labeled base molds filled with frozen tissue matrix (OCT®). Sections of 25 μm were cut at -20°C by using a cryostat (Leica CM 1850, Leica Biosystem, Germany). The sections obtained were stored in PBS at 4 ^o^C for overnight, following removal of PBS, added cryoprotectant solution and stored at -20 ^o^C until further use for Nissl and glial staining.^23^ Histology was performed on hippocampus region of the brain to correlate the differences in spatial, memory, behavioral and cognitive learning. The hippocampal region was identified according to the Mouse Brain Atlas Map,^24^ and we screened 3-4 sections/animal (11 control and 12 NP treated animals) to provide sufficient replicates to detect a significant difference at a power analysis of 0.8. Image J software (Version 1. 7) was used to determine neuronal and glial counts in the hippocampus from Nissl and anti-TMEM119 stained sections respectively.

*Nissl staining*

Nissl staining was performed to evaluate the neuronal density. The cryopreserved sections were washed with PBS, and incubated with Nissl Staining Solution (10 ml of 0.1% Cresyl violet solution) for 20 min at room temperature. Sections were then dehydrated with 95% and 100% ethanol, washed with xylene and added 1 drop of Dibutylphthalate Polystyrene Xylene (DPX) mounting media on glass slide, and covered with a thin glass coverslip imaged using microscope (DM5500 B, Leica Microsystems, Germany).

*Immunohistochemistry – Microglial cell immunostaining*

Immunostaining was performed to count microglial cells following standard company protocols (Cat. No. ab209064). Cryopreserved brain sections (25μm) were washed with 1xPBS three times (10 min each) and the sections were incubated with 0.3% hydrogen peroxide for 10 min at room temperature to remove the endogenous peroxidase activity. The sections were transferred to the blocking buffer (1% BSA dissolved in PBST (1xPBS+0.1% Triton X-100)) and kept at room temperature for one hour. Followed by the blocking, sections were incubated with *anti*-rabbit-TMEM-119 primary antibody (1:500; Abcam, USA) diluted in blocking buffer and incubated at 4°C for overnight. Subsequently, sections were then washed in PBST thrice (5 min each) and incubated with goat *anti*-rabbit IgG-HRP secondary antibody (1:2000; Abcam, USA) diluted in blocking buffer at room temperature for 1h and washings repeated. Following washings, 3,3’ diaminobenzidine (DAB; mouse and rabbit specific HRP/DAB detection IHC kit) was added; the sections were then washed with PBS and mounted by using DPX mounting media on slides and imaged using microscope (DM5500 B, LICA, Germany).

*Collection of fecal samples and isolation of fecal microbial DNA*

At 30, 60, 90, and 120 days of age, each animal was placed in a cage alone without any bedding. Four to five fecal boli were collected from each animal and placed in sterile 2 mL cryogenic vials (Corning Incorporated, Corning, NY) and placed in liquid N_2_ until they were transferred to a -80℃ freezer. Thereafter, they were stored until being used for bacterial isolation and gut microbiota analysis. The fecal microbial DNA was isolated from a portion of the fecal boli collected using the Invitrogen Pure Link Microbiome DNA Purification Kit (Thermo Fisher Scientific, Waltham, MA) and in accordance with the manufacturer’s protocol. The quantity of DNA isolated was measured using Qubit 3.0 Fluorometer (Life Technologies, Grand Island, NY). The number of replicates (**Table S1**) tested is comparable to other studies examining how *in utero* environmental changes can affect gut bacterial populations and have shown that such sample sizes can result in statistical differences between offspring groups ^25, 26^.

*16S rRNA sequencing*

The University of Missouri (MU) DNA Core Facility prepared bacterial 16S ribosomal DNA amplicons from extracted fecal DNA by amplification of the V4 hypervariable region of the 16S rDNA with universal primers (U515F/806R) flanked by Illumina standard adapter sequences.^27, 28^ The rest of the procedures were performed as described previously.^29, 30^ The resulting amplicon pool was analyzed by using the Advanced Analytical Fragment Analyzer automated electrophoresis system, quantified with a Qubit fluorometer using a quant-iT HS dsDNA reagent kit (Invitrogen), and diluted according to Illumina’s standard protocol for sequencing on the MiSeq.

**SUPPLEMENTARY FIGURES**


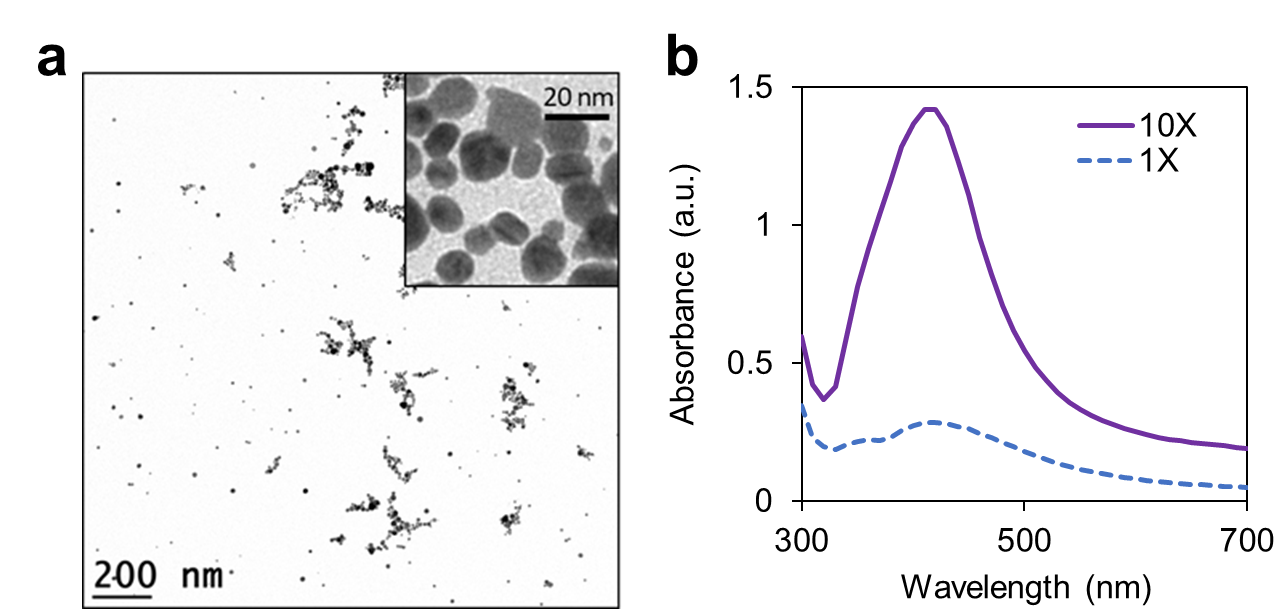


Figure S1. Characterization of AgNPs: (a) TEM image and (b) UV-Visible absorption spectra. Representative images from triplicate studies.


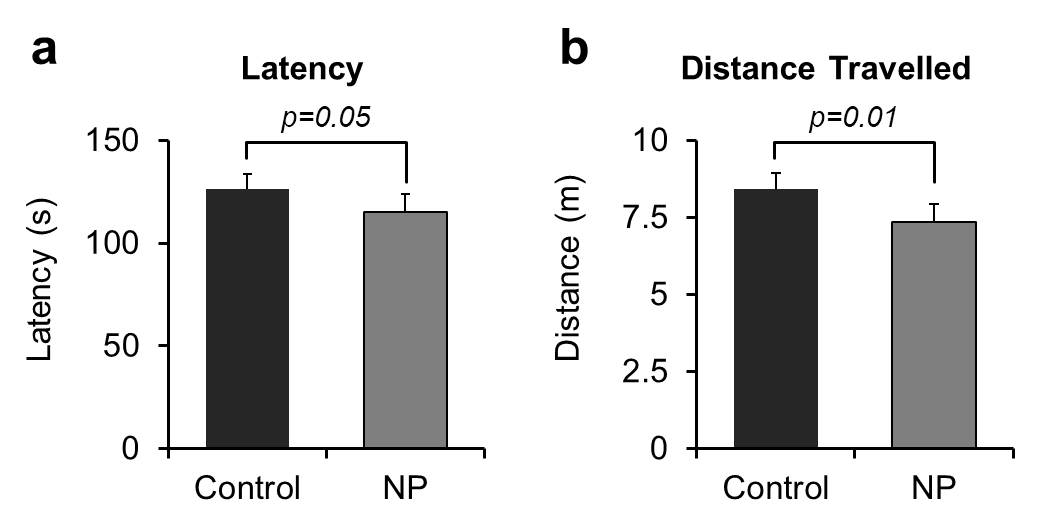


Figure S2. Barnes Maze results for: (a) Latency and, (b) Distance travelled. *p* value differences are designated above the graphs. Replicates tested include n=11 for Ctrl; n=12 for NP.


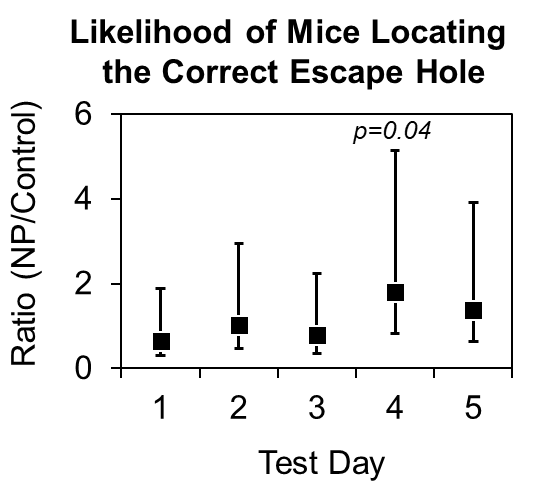


Figure S3. Likelihood of mice locating the correct escape hole. *p* value differences are designated above the graphs. Replicates tested include n=11 for Ctrl; n=12 for NP.


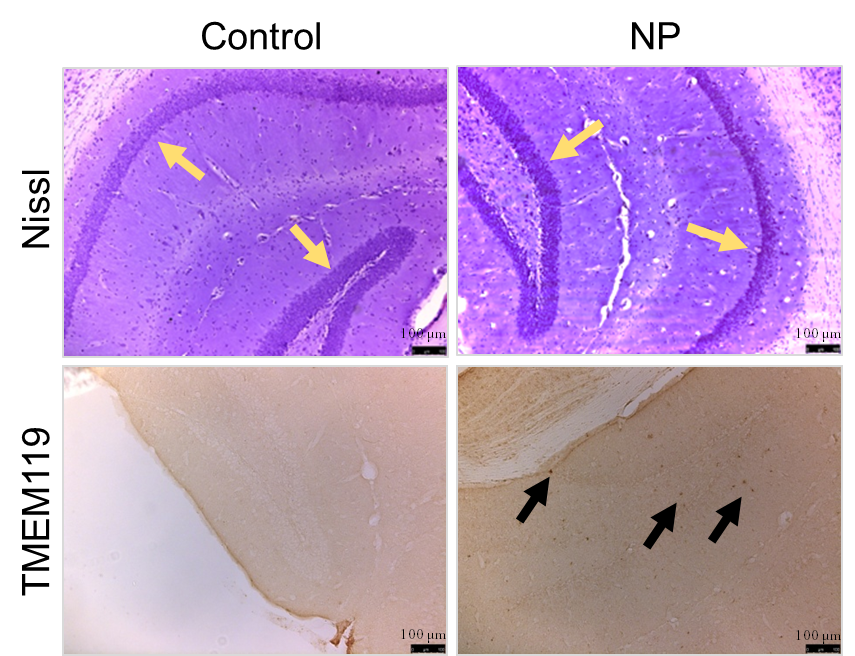


Figure S4. Representative brain sections containing hippocampus stained with Nissl dye for neurons and *anti*-TMEM-119 for microglial cells. Yellow and black arrows represent neurons and microglial cells respectively. Scale bar represents 100 µm.


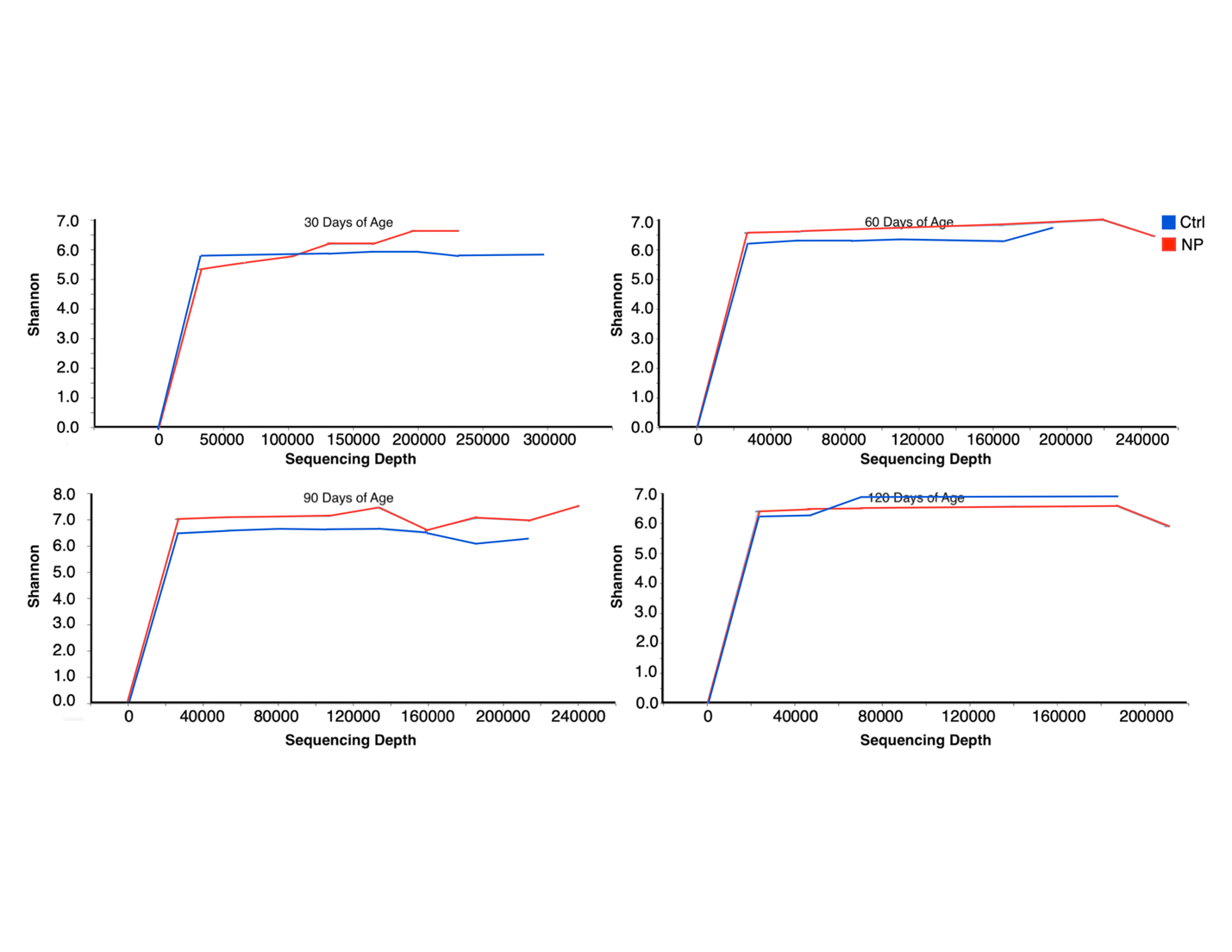


Figure S5. Alpha-diversity based on Shannon analysis. Replicates tested include Ctrl and AgNP exposed groups respectively at 30 (n=10 and n =9), 60 (n=11 and n=10), 90 (n=12 and n =10), and 120 (n=10 and n =12) days of age.


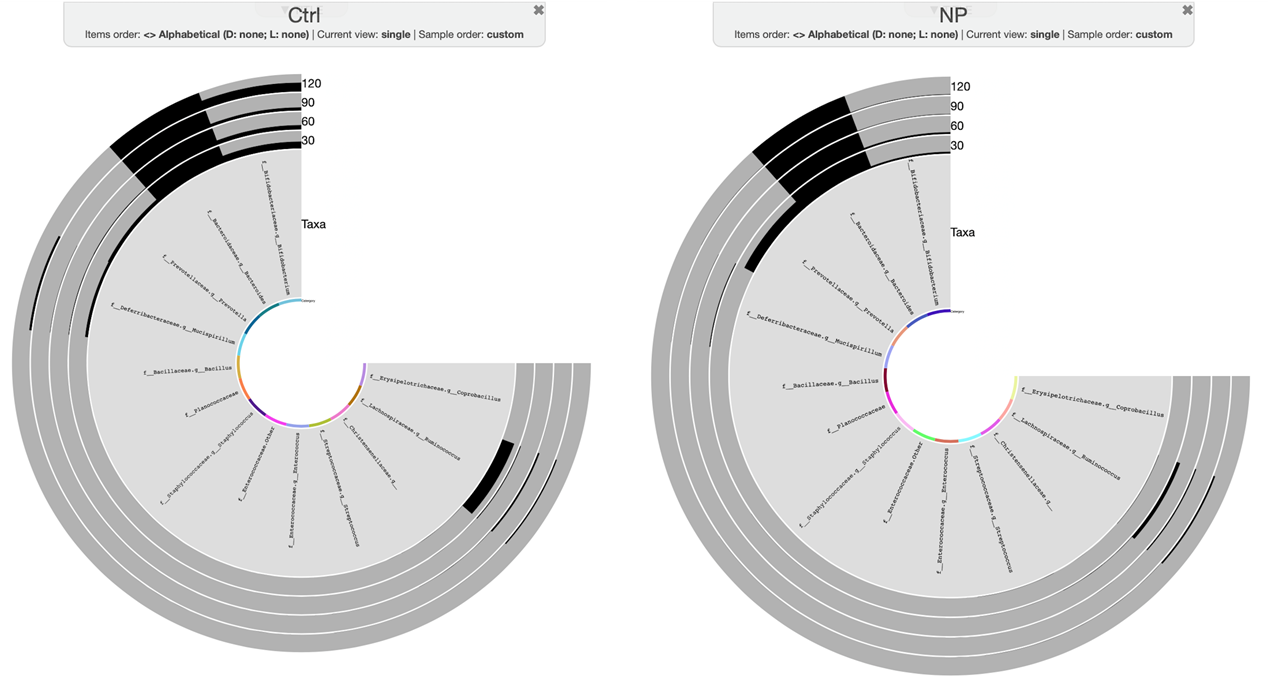


Figure S6. Anvi’o diagram of bacterial changes over time. The figure shows the expression of these bacteria. Replicates tested include Ctrl and AgNP exposed groups respectively at 30 (n=10 and n =9), 60 (n=11 and n=10), 90 (n=12 and n =10), and 120 (n=10 and n =12) days of age.

**SUPPLEMENTARY TABLES**

Table S1. Number of replicates used for the gut microbiome study. The same animals were tested at each age, but low reads resulted in exclusion of select samples for a given age.

|  | *Sex* | | *Treatment* | |
| --- | --- | --- | --- | --- |
| *Age (Days)* | *Male* | *Female* | *Ctrl* | *NP* |
| *30* | 10 | 9 | 10 | 9 |
| *60* | 12 | 10 | 11 | 11 |
| *90* | 11 | 11 | 12 | 10 |
| *120* | 11 | 11 | 10 | 12 |

Table S2. Other EPM behavioral assessments in the control and nanoparticle treated groups. Replicates tested include n=12 for AgNP exposed group; n=11 for Ctrl.

|  |  | *Control* | | *Nanoparticle* | |
| --- | --- | --- | --- | --- | --- |
| *Item* |  | *Mean* | *SEM* | *Mean* | *SEM* |
| *Center* | *Rate*  *(m/sec)* | 2.34 | 0.15 | 2.44 | 0.28 |
|  | *Duration*  *(sec)* | 116.76 | 8.21 | 126.75 | 7.67 |
| *Closed arms* | *Rate*  *(m/sec)* | 2.34 | 0.22 | 2.00 | 0.19 |
|  | *Duration*  *(sec)* | 188.67 | 13.30 | 164.12 | 18.81 |
| *Mobile* | *Rate*  *(m/sec)* | 0.41 | 0.11 | 0.33 | 0.07 |
|  | *Duration*  *(sec)* | 151.64 | 37.07 | 167.23 | 34.00 |
| *Open arms* | *Rate*  *(m/sec)* | 0.08 | 0.04 | 0.11 | 0.05 |
|  | *Duration*  *(sec)* | 3.43 | 1.52 | 3.33 | 1.47 |

Table S3. Other echo-MRI results for Ctrl and NP. Replicates tested include n=11 for Ctrl and n=12 for AgNP exposed group.

|  | *Control* | |  | *Nanoparticle* | |
| --- | --- | --- | --- | --- | --- |
| *Item* | *Mean (g)* | *SEM* | *Significant P value* | *Mean (g)* | *SEM* |
| *Lean* | 19.62 | 0.56 |  | 20.51 | 0.73 |
| *Free Water* | 0.07 | 0.02 |  | 0.08 | 0.02 |
| *Weight* | 24.98 | 1.03 |  | 26.78 | 1.60 |
| *Fat Percentage* | 16.05 | 1.26 | 0.01 | 21.85 | 2.77 |
| *Lean Percentage* | 79.04 | 1.59 |  | 78.10 | 2.87 |
| *Free Water Percentage* | 0.27 | 0.07 |  | 0.32 | 0.08 |
| *Total Water Percentage* | 64.17 | 1.33 |  | 63.84 | 2.40 |

***References***

1. Tolaymat, T. M.; El Badawy, A. M.; Genaidy, A.; Scheckel, K. G.; Luxton, T. P.; Suidan, M., An evidence-based environmental perspective of manufactured silver nanoparticle in syntheses and applications: A systematic review and critical appraisal of peer-reviewed scientific papers. *Science of The Total Environment* **2010,** *408* (5), 999-1006.

2. Javurek, A. B.; Suresh, D.; Spollen, W. G.; Hart, M. L.; Hansen, S. A.; Ellersieck, M. R.; Bivens, N. J.; Givan, S. A.; Upendran, A.; Kannan, R.; Rosenfeld, C. S., Gut Dysbiosis and Neurobehavioral Alterations in Rats Exposed to Silver Nanoparticles. *Sci Rep* **2017,** *7* (1), 2822.

3. Wu, J.; Yu, C.; Tan, Y.; Hou, Z.; Li, M.; Shao, F.; Lu, X., Effects of prenatal exposure to silver nanoparticles on spatial cognition and hippocampal neurodevelopment in rats. *Environ Res* **2015,** *138*, 67-73.

4. Austin, C. A.; Hinkley, G. K.; Mishra, A. R.; Zhang, Q.; Umbreit, T. H.; Betz, M. W.; B, E. W.; Casey, B. J.; Francke-Carroll, S.; Hussain, S. M.; Roberts, S. M.; Brown, K. M.; Goering, P. L., Distribution and accumulation of 10 nm silver nanoparticles in maternal tissues and visceral yolk sac of pregnant mice, and a potential effect on embryo growth. *Nanotoxicology* **2016,** *10* (6), 654-61.

5. Austin, C. A.; Umbreit, T. H.; Brown, K. M.; Barber, D. S.; Dair, B. J.; Francke-Carroll, S.; Feswick, A.; Saint-Louis, M. A.; Hikawa, H.; Siebein, K. N.; Goering, P. L., Distribution of silver nanoparticles in pregnant mice and developing embryos. *Nanotoxicology* **2012,** *6*, 912-22.

6. Charehsaz, M.; Hougaard, K. S.; Sipahi, H.; Ekici, A. I.; Kaspar, C.; Culha, M.; Bucurgat, U. U.; Aydin, A., Effects of developmental exposure to silver in ionic and nanoparticle form: A study in rats. *Daru* **2016,** *24* (1), 24.

7. Lee, Y.; Choi, J.; Kim, P.; Choi, K.; Kim, S.; Shon, W.; Park, K., A transfer of silver nanoparticles from pregnant rat to offspring. *Toxicol Res* **2012,** *28* (3), 139-41.

8. Melnik, E. A.; Buzulukov, Y. P.; Demin, V. F.; Demin, V. A.; Gmoshinski, I. V.; Tyshko, N. V.; Tutelyan, V. A., Transfer of Silver Nanoparticles through the Placenta and Breast Milk during in vivo Experiments on Rats. *Acta Naturae* **2013,** *5* (3), 107-15.

9. Philbrook, N. A.; Winn, L. M.; Afrooz, A. R.; Saleh, N. B.; Walker, V. K., The effect of TiO(2) and Ag nanoparticles on reproduction and development of Drosophila melanogaster and CD-1 mice. *Toxicol Appl Pharmacol* **2011,** *257* (3), 429-36.

10. Wang, Z.; Qu, G.; Su, L.; Wang, L.; Yang, Z.; Jiang, J.; Liu, S.; Jiang, G., Evaluation of the biological fate and the transport through biological barriers of nanosilver in mice. *Curr Pharm Des* **2013,** *19* (37), 6691-7.

11. Kovvuru, P.; Mancilla, P. E.; Shirode, A. B.; Murray, T. M.; Begley, T. J.; Reliene, R., Oral ingestion of silver nanoparticles induces genomic instability and DNA damage in multiple tissues. *Nanotoxicology* **2015,** *9* (2), 162-71.

12. Yu, W. J.; Son, J. M.; Lee, J.; Kim, S. H.; Lee, I. C.; Baek, H. S.; Shin, I. S.; Moon, C.; Kim, S. H.; Kim, J. C., Effects of silver nanoparticles on pregnant dams and embryo-fetal development in rats. *Nanotoxicology* **2014,** *8 Suppl 1*, 85-91.

13. Ghaderi, S.; Tabatabaei, S. R. F.; Varzi, H. N.; Rashno, M., Induced adverse effects of prenatal exposure to silver nanoparticles on neurobehavioral development of offspring of mice. *The Journal of Toxicological Sciences* **2015,** *40* (2), 263-275.

14. Ferdous, Z.; Nemmar, A., Health Impact of Silver Nanoparticles: A Review of the Biodistribution and Toxicity Following Various Routes of Exposure. *Int J Mol Sci* **2020,** *21* (7).

15. Morishita, Y.; Yoshioka, Y.; Takimura, Y.; Shimizu, Y.; Namba, Y.; Nojiri, N.; Ishizaka, T.; Takao, K.; Yamashita, F.; Takuma, K.; Ago, Y.; Nagano, K.; Mukai, Y.; Kamada, H.; Tsunoda, S.-i.; Saito, S.; Matsuda, T.; Hashida, M.; Miyakawa, T.; Higashisaka, K.; Tsutsumi, Y., Distribution of Silver Nanoparticles to Breast Milk and Their Biological Effects on Breast-Fed Offspring Mice. *ACS Nano* **2016,** *10* (9), 8180-8191.

16. Alomaim, H.; Griffin, P.; Swist, E.; Plouffe, L. J.; Vandeloo, M.; Demonty, I.; Kumar, A.; Bertinato, J., Dietary calcium affects body composition and lipid metabolism in rats. *PloS one* **2019,** *14* (1), e0210760-e0210760.

17. Attar, A.; Liu, T.; Chan, W.-T. C.; Hayes, J.; Nejad, M.; Lei, K.; Bitan, G., A shortened Barnes maze protocol reveals memory deficits at 4-months of age in the triple-transgenic mouse model of Alzheimer's disease. *PloS one* **2013,** *8* (11), e80355-e80355.

18. Johnson, S. A.; Javurek, A. B.; Painter, M. S.; Ellersieck, M. R.; Welsh, T. H.; Camacho, L.; Lewis, S. M.; Vanlandingham, M. M.; Ferguson, S. A.; Rosenfeld, C. S., Effects of developmental exposure to bisphenol A on spatial navigational learning and memory in rats: A CLARITY-BPA study. *Hormones and behavior* **2016,** *80*, 139-148.

19. Jašarević, E.; Sieli, P. T.; Twellman, E. E.; Welsh, T. H., Jr.; Schachtman, T. R.; Roberts, R. M.; Geary, D. C.; Rosenfeld, C. S., Disruption of adult expression of sexually selected traits by developmental exposure to bisphenol A. *Proc Natl Acad Sci U S A* **2011,** *108* (28), 11715-20.

20. Bedrosian, T. A.; Herring, K. L.; Weil, Z. M.; Nelson, R. J., Altered temporal patterns of anxiety in aged and amyloid precursor protein (APP) transgenic mice. *Proc Natl Acad Sci U S A* **2011,** *108* (28), 11686-91.

21. Harris, J. A.; Devidze, N.; Halabisky, B.; Lo, I.; Thwin, M. T.; Yu, G. Q.; Bredesen, D. E.; Masliah, E.; Mucke, L., Many neuronal and behavioral impairments in transgenic mouse models of Alzheimer's disease are independent of caspase cleavage of the amyloid precursor protein. *J Neurosci* **2010,** *30* (1), 372-81.

22. Abu Eid, S.; Hackl, M. T.; Kaplanian, M.; Winter, M.-P.; Kaltenecker, D.; Moriggl, R.; Luger, A.; Scherer, T.; Fürnsinn, C., Life Under Hypoxia Lowers Blood Glucose Independently of Effects on Appetite and Body Weight in Mice. *Frontiers in endocrinology* **2018,** *9*, 490.

23. Chen, D.; Nie, Z. B.; Chi, Z. H.; Wang, Z. Y.; Wei, X. T.; Guan, J. H., Neuroprotective Effect of ZnT3 Knockout on Subarachnoid Hemorrhage. *Transl Neurosci* **2018,** *9*, 26-32.

24. Paxinos, G.; Franklin, K. B. J., *The mouse brain in stereotaxic coordinates*. 2019.

25. Ma, J.; Prince, A. L.; Bader, D.; Hu, M.; Ganu, R.; Baquero, K.; Blundell, P.; Alan Harris, R.; Frias, A. E.; Grove, K. L.; Aagaard, K. M., High-fat maternal diet during pregnancy persistently alters the offspring microbiome in a primate model. *Nat Commun* **2014,** *5*, 3889.

26. Thorburn, A. N.; McKenzie, C. I.; Shen, S., Evidence that asthma is a developmental origin disease influenced by maternal diet and bacterial metabolites. *Nat Commun* **2015,** *6*, 7320.

27. Caporaso, J. G.; Lauber, C. L.; Walters, W. A.; Berg-Lyons, D.; Lozupone, C. A.; Turnbaugh, P. J.; Fierer, N.; Knight, R., Global patterns of 16S rRNA diversity at a depth of millions of sequences per sample. *Proc Natl Acad Sci U S A* **2011,** *108 Suppl 1*, 4516-22.

28. Walters, W. A.; Caporaso, J. G.; Lauber, C. L.; Berg-Lyons, D.; Fierer, N.; Knight, R., PrimerProspector: de novo design and taxonomic analysis of barcoded polymerase chain reaction primers. *Bioinformatics* **2011,** *27* (8), 1159-61.

29. Javurek, A. B.; Spollen, W. G.; Johnson, S. A.; Bivens, N. J.; Bromert, K. H.; Givan, S. A.; Rosenfeld, C. S., Effects of exposure to bisphenol A and ethinyl estradiol on the gut microbiota of parents and their offspring in a rodent model. *Gut Microbes* **2016,** *7* (6), 471-485.

30. Koestel, Z. L.; Backus, R. C.; Tsuruta, K.; Spollen, W. G.; Johnson, S. A.; Javurek, A. B.; Ellersieck, M. R.; Wiedmeyer, C. E.; Kannan, K.; Xue, J.; Bivens, N. J.; Givan, S. A.; Rosenfeld, C. S., Bisphenol A (BPA) in the serum of pet dogs following short-term consumption of canned dog food and potential health consequences of exposure to BPA. *Sci Total Environ* **2017,** *579*, 1804-1814.
